# Concomitant sentinel lymph node biopsy leading to abbreviated systematic lymphadenectomy in a patient with primary malignant melanoma of the vagina

**DOI:** 10.1186/s40064-014-0773-x

**Published:** 2015-02-28

**Authors:** Hiroaki Ishida, Tomonori Nagai, Syo Sato, Michiko Honda, Takahiro Uotani, Kouki Samejima, Tatsuya Hanaoka, Taichi Akahori, Yasushi Takai, Hiroyuki Seki

**Affiliations:** Saitama Medical Center Japan, Obstetrics and Gynecology, 1981 Kamoda, Kawagoe City, Saitama 350-8550 Japan

**Keywords:** Primary malignant melanoma of the vagina, Sentinel-node biopsy, Vaginal cancer

## Abstract

**Introduction:**

Primary malignant melanoma of the vagina is an extremely rare disease affecting 3% of patients with malignant vaginal tumors. It is rare compared to primary malignant melanoma of the skin and its prognosis is unfavorable even in patients with Stage I disease. Here, we report a case of primary malignant melanoma of the vagina and discuss our experience with regard to previously published literature.

**Case description:**

The patient was a 59-year-old female with 2 prior pregnancies and child births. She was examined by a local doctor for swelling of the genitalia, and a 1.8 × 1.0 cm large tumor was detected on the left side of the vaginal wall. A biopsy indicated leiomyosarcoma, and she was referred to our hospital. The tumor was resected, and histopathology of the resected sample confirmed the diagnosis of malignant melanoma based on a positive surgical margin. Additional courses of treatment included left inguinal sentinel lymph node biopsy using an isotope and extended vaginectomy. Although the sentinel node was negative, we performed a modified radical hysterectomy and left vaginectomy during the third operation because the surgical margin was positive. We could not confirm whether the lesion in the extracted sample was malignant, and the final diagnosis was primary malignant melanoma of the vagina T4bN0M0 Stage IIc (UICC 2009). Postoperative adjuvant therapy consisted of 6 cycles of DAV-Feron therapy (dacarbazine, ACNU, vincristine, IFN- β). After 5 months of postoperative adjuvant therapy, a 2 cm single lung metastasis was detected in the lower left lung. We performed a laparoscopic lower left lobectomy and are planning additional chemotherapy.

**Discussion and evaluation:**

Currently, surgical resection has the highest probability of improving the prognosis of patients when used as initial treatment for Stage I disease. By combining treatment with sentinel lymph node biopsy, we were able to accurately determine the stage of disease and thus avoid systematic lymph node dissection and further surgical treatments.

**Conclusion:**

Malignant melanoma of the vagina is very rare tumor so it is necessary to requires the integration of further cases.

## Introduction

Primary malignant melanoma of the vagina is an extremely rare condition that affects 1% of women with malignant melanoma and less than 3% of women with malignant tumors of the vagina (Piura et al. 2002; Gokaslan et al. 2005; Nakagawa et al. 2002).

Compared to normal malignant tumors of the skin, hematogenous and lymphogenous metastasis occurs more frequently in malignant melanoma of the vagina.and unfavorable prognosis even in early stage disease (Frumovitz et al. 2010). We report our experience with a patient who presented with primary malignant melanoma and discuss our findings with regard to previously published literature.

## Case description

**Figure 1:**
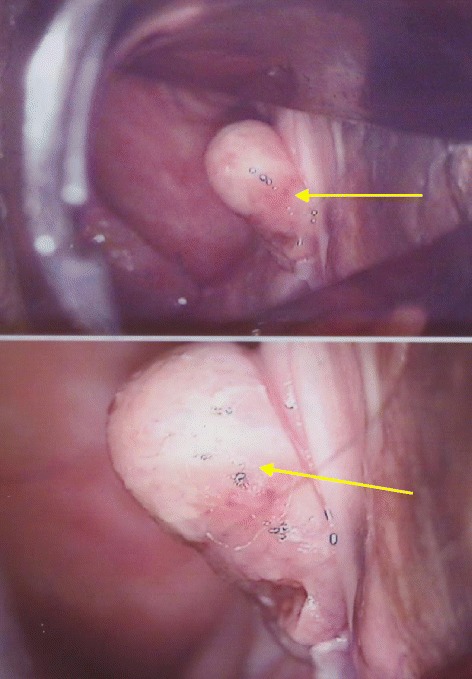
**We confirmed a white, vaginal wall tumor 1.8 × 1.0 cm in size on the left side of the vagina, 1 cm from the vaginal opening, by speculum examination.**

The patient was a 59-year-old female with 2 prior pregnancies and child births and no particular prior medical or family history. In January 2012, she noticed swelling in the genitals and sought the advice of a local obstetrician/gynecologist. A tumor measuring 2 cm was detected on the left side 1 cm from the vaginal opening. She was diagnosed with a tumor of the vaginal wall (spindle cell tumor) based on the results of biopsy and histopathology, and she was referred to our clinic. We confirmed the presence of a white, vaginal wall tumor 1.8 × 1.0 cm in size (Figure 1) located on the left side, 1 cm from the vaginal opening by speculum examination. No abnormalities in the initial blood biochemistry or tumor marker values were detected. Laboratory values were as follows: WBC, 5200/μl; RBC, 437/μl; Hb, 13.6 g/dl; Plt, 20.4 × 10^4^/μl; LDH, 195 μl; CRP, 0.0 mg/dl; CEA, 3.2 ng/ml; CA125, 14 U/ml; CA19-9, 21 U/ml; and SCC, 0.7 ng/ml.

**Figure 2:**
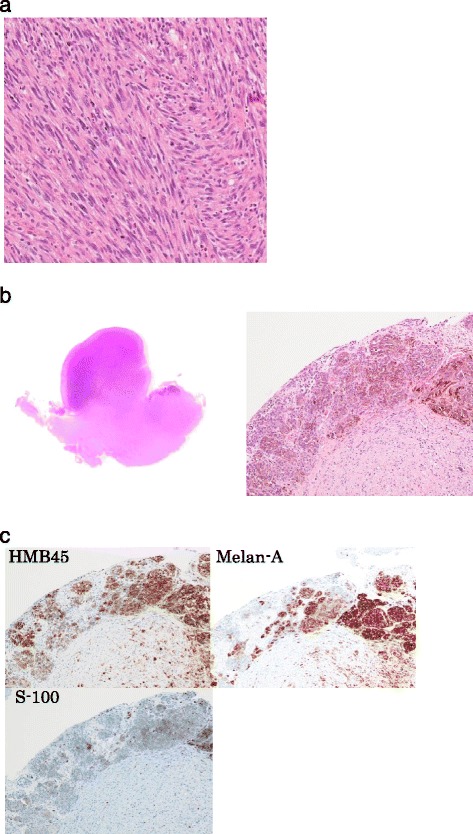
**Vaginal wall tumor resection. (a)** Diagnosis of spindle cell tumor suspicious of leiomyosarcoma. (H&E100×) **(b)** tumor thickness of 8.5 mm and brown pigmentation in the cytoplasm (H&E 1×,40×) **(c)** immunostaining revealed that the tumor cells were Positive for S-100 protein, HMB45, and Melan-A (40×).

Histological diagnosis: spindle cell tumor with suspicion of leiomyosarcoma (Figure 2a).

No clear evidence of distant metastasis or vaginal wall tumors was obtained by CT examination or pelvic MRI.

Histopathological diagnosis performed using specimens from the previous doctor indicated spindle cell tumor suspicious of leiomyosarcoma, and a vaginal wall tumor resection was qa 1 cm surrounding margin. Histopathology of the resected sample indicated a malignant melanoma-positive surgical margin (Figure 2b, 2c).

**Figure 3:**
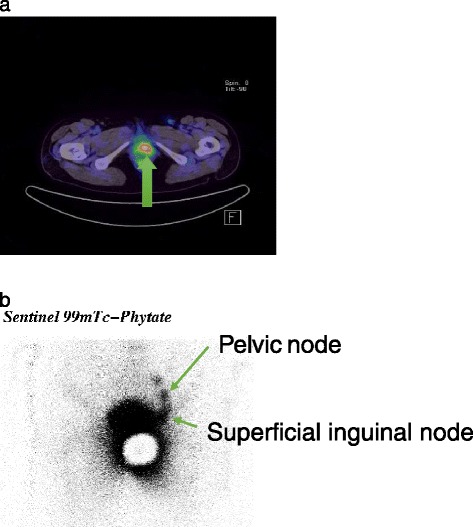
**PET examination and nuclear medicine scan (a) high accumulation of FDG was confirmed in the left side of the vagina (arrow) (b) Accumulaion of m99Tc in the left inguinal to retroperitoneal lymph nodes (arrow).**

An FDG-PET-CT scan was performed after the first operation to rule out the presence of remaining lesions or distant metastasis. The results showed an area of high FDG accumulation on the left side of the vagina; however, tracer accumulation in the lymph nodes or evidence of distant metastasis was not found (Figure 3a).

In preparation for additional surgery including sentinel lymph node biopsy, we injected m99Tc into the region of the vaginal wall tumor 1 day before surgery, and confirmed the accumulation of m99Tc 2 hours later in the left inguinal to retroperitoneal lymph node (Figure 3b).

A second surgery was performed 35 days after the first operation. We found surgical scars from the previous operation 1 cm from the vaginal opening on the left side. Therefore, the tumor was resected with a 2 cm margin and resection of the vaginal wall was performed. A sentinel lymph node (left superficial inguinal lymph node) biopsy was performed using an isotope, which did not detect lymph node metastasis (0/3) in a rapid histopathological examination. Therefore, pelvic and inguinal lymph node metastasis was not performed.

Histopathological analysis of the resected samples revealed malignant melanoma, and a microscopic distance of 0.3 mm was observed between the resected margin of the vaginal wall and the tumor. This indicated that the resected region did not fall within the safety margin and an additional surgery was scheduled. A third surgery was performed 35 days after the second operation. To ensure adequate vaginal wall resection, we performed an abdominal modified radical hysterectomy followed by a left-side vaginectomy.

Surgical findings: We performed an abdominal modified radical hysterectomy. After processing the anterior layer of the vesico-uterine ligament bilaterally, we separated the bladder from the vaginal wall from the outside of the left abdominal cavity. We then processed the paravaginal tissue on the left side by accessing it from the abdominal side; vaginal wall resection was performed by approaching it from the external genitalia. Finally, we penetrated the abdominal cavity and the left side of the vagina, and removed the left side of the vagina and the uterus together.

**Figure 4:**
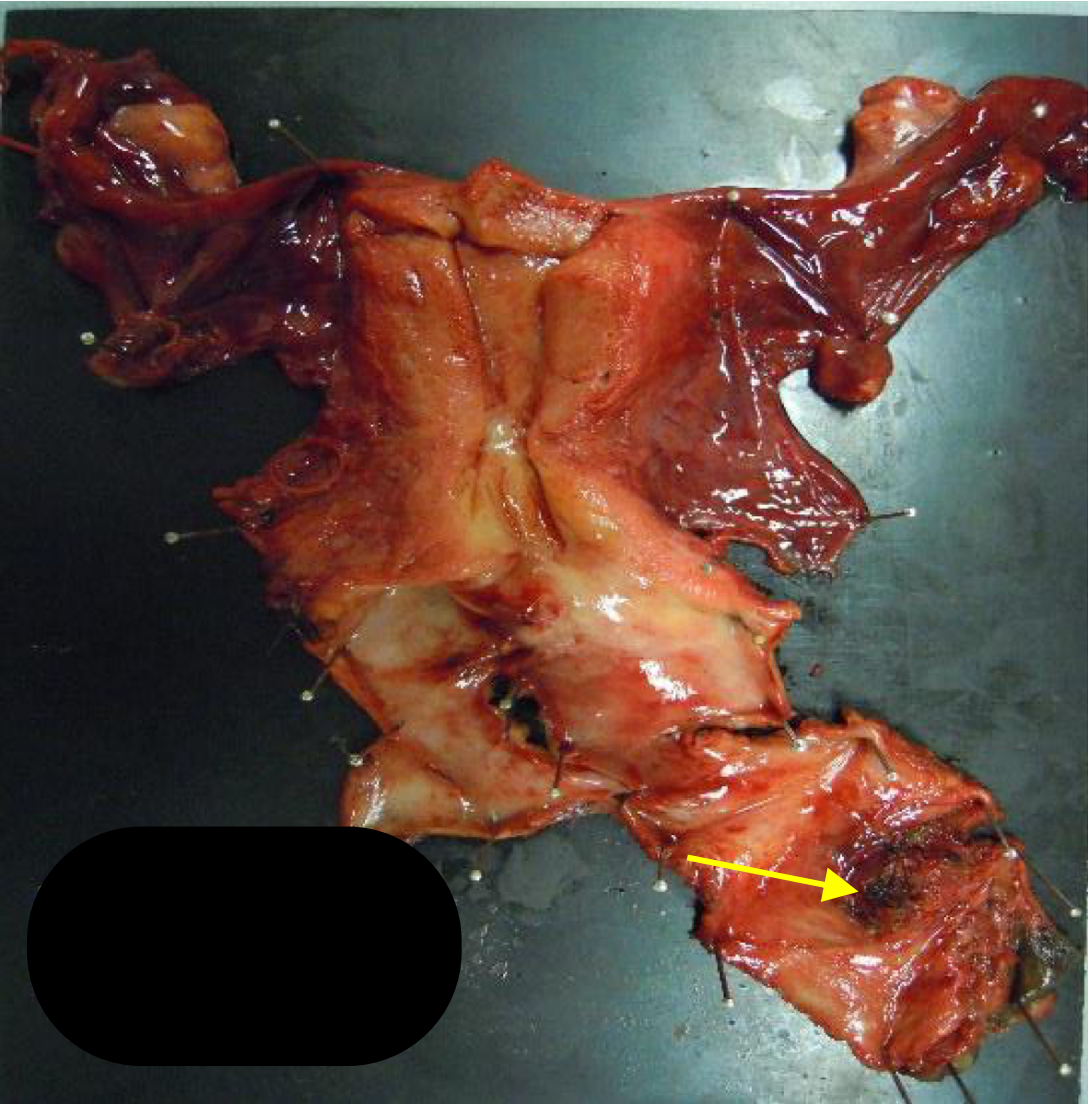
**Uterine-bladder sample from the third surgery (macro).** The scar from the previous surgery could be confirmed with the naked eye (arrow).

A modified radical hysterectomy was selected instead of a simple total hysterectomy to ensure adequate excision of the vaginal wall by separating it from the ureter. Moreover, we excised the vesical branches of the pelvic splanchnic nerve on the left side to ensure adequate separation of the bladder from the vaginal wall on the left side. We did not opt for total resection of the vaginal wall, as a similar approach from the right would have ensured post-operative dysuria. Instead, we limited the operation to the left side. The total time required for the procedure was 8 hours 30 minutes with a blood loss of 1440 ml. As shown in the analysis of the extracted sample (Figure 4), we found no evidence of remaining malignant tissue.

The final diagnosis was primary malignant melanoma of the vagina T4bN0M0 Stage IIc (UICC 2009), based on the tumor size of 2 cm, over 4 mm of interstitial invasion, the lack of evidence of metastasis to the inguinal lymph nodes extracted as sentinel lymph nodes, and the lack of evidence of distant metastasis.

A 6-course postoperative adjuvant therapy with DAV-Feron was planned (Table 1). Four courses of DAV-Feron treatment were administered between June and November 2012. Although a fifth course of therapy was planned, the patient developed grade 4 thrombocytopenia and the treatment was changed to 2 courses of locally injected Feron-only therapy. At 5 months after terminating the adjuvant therapy (18 months after the initial treatment), a 2 cm single lung metastasis was detected in the lower-left lung field. It was diagnosed as recurrence of the malignant melanoma of the vaginal wall metastasized to the lungs, and we performed a laparoscopic lower left lobectomy. Analysis of the resected sample revealed relapsed malignant melanoma with a negative surgical margin. Additional chemotherapy is currently being planned.

**Table 1:** **DAV-Feron chemotherapy**

	**day1**	**day2**	**day3**	**day4**	**day 5**
DTIC: 120 mg/m2/day, iv.	↓	↓	↓	↓	↓
ACNU: 60 mg/m2/day, iv.	↓				
VCR: 0.6 mg/m2/day, iv	↓				
Feron: 3,000,000 E/body/day, local injection	↓	↓	↓	↓	↓

Every 4-6 weeks, total 5-6 courses.

DTIC: Dacarvazine, ACNU : Nimustine hydrochloride, VCR : Vincristine, Feron : INF-β.

↓: Chemotherapy is performed.

## Discussion

Primary malignant melanoma of the vagina is an extremely rare condition that affects 1% of women with malignant melanoma and less than 3% of women diagnosed with malignant tumors of the vagina. Approximately 250 cases have been reported in the literature and 0.026 in 100,000 people are expected to present with this condition each year (Piura et al. 2002; Gokaslan et al. 2005; Nakagawa et al. 2002). Approximately 3% of females have melanocytes (melanin-producing pigment cells) in the basement membrane of the epithelium of the vaginal mucous membrane, which can be the site of onset of primary malignant melanoma of the vagina (Gokaslan et al. 2005). Approximately 80% of cases occur post-menopause with an average age of onset of 60 years, and the primary symptom is vaginal bleeding (Frumovitz et al. 2010). A study showed that 84% of patients are Caucasian (Frumovitz et al. 2010). Most cases occur in the lower third of the vagina on the anterior vaginal wall (Nakagawa et al. 2002). Although malignant melanoma often presents with pigmentation, our patient was amelanotic. Reports suggest that amelanotic cases account for less than 10% of all cases (Nakagawa et al. 2002).

In the determination of the clinical stage of primary malignant melanoma of the vagina, the staging classification of the International Federation of Gynecology and Obstetrics (FIGO), which is used in vaginal cancers, does not combine tumor size and lymph node metastasis. Instead, staging of primary malignant melanoma of the vagina is based on the classification of the Union for International Cancer Control 2009 (UICC), which is used for the staging of malignant melanoma.

Several advanced cases of primary malignant melanoma of the vagina have been reported, and the prognosis is extremely poor even in early stage disease. Frumovitz et al. reported that the 5-year survival rate for Stage I primary malignant melanoma of the skin is 81% compared to 18% for melanoma originating in the vagina. The authors analyzed 37 patients with Stage I primary malignant melanoma of the vagina and reported a 5-year progression-free survival rate of 9.5% and a 5-year overall survival rate of 20.0% (Frumovitz et al. 2010).

Frumovitz et al. reported on the effectiveness of surgical therapies for primary malignant melanoma of the vagina. These authors found that patients treated with only radiation or chemotherapy as initial treatment for Stage I primary malignant melanoma of the skin had an average survival of 8.7 months, compared to 24–32 months for those that also received prior surgical therapies, which was a significant increase (P = 0.01) in survival (Frumovitz et al. 2010).

However, with respect to the surgical methods, they reported no significant differences between patients who underwent lesion excision, radical surgery, or pelvic exenteration (Frumovitz et al. 2010). Pelvic exenteration often presents with surgery-related complications, which prevents it from becoming a standard therapy, especially if adjuvant therapies are considered. The definitiveness of local excision of lesions can be a problem in addition to the possibility of local recurrence associated with this surgical method.

Therapeutic considerations such as the systematic removal of inguinal and pelvic lymph nodes in primary malignant melanoma of the vagina are uncommon; however, lymph node metastasis is a prognostic factor. Therefore, the detection of lymph node metastasis is necessary. However, considering the invasiveness of surgical intervention, operations should be limited as much as possible. In general, sentinel lymph node biopsies are indicated in patients with malignant melanoma and are used frequently in other primary sites (Lens et al. 2002). The MSLT-1 study investigated the effectiveness of sentinel lymph node biopsies, and we performed sentinel lymph node biopsies based on this theoretical background.

To determine whether sentinel lymph node biopsies improved survival rates in all cases of malignant melanoma, a randomized, comparative trial was conducted in collaboration with 17 institutions (MSLT-1). In this trial, 1269 patients with primary tumor thicknesses between 1.2 mm and 3.5 mm were divided into 2 groups consisting of 769 patients receiving sentinel lymph node biopsies and 500 patients receiving primary lesion resection only (dissection was performed after discovery of lymph node metastasis during post-operative periodic observations). The results showed that the 5-year progression free survival rate in patients with biopsies was 78.3 ± 1.3% compared to 73.1 ± 2.1% for those without, which was a significant increase in the former group (P = 0.009, death HR: 0.74) (Morton et al. 2006).

Our study also demonstrated that lymph node dissection should be performed early if sentinel lymph node biopsies reveal metastasis by microscopy, instead of waiting until metastasis is observed during periodic observation after primary tumor resection. However, whether lymph node dissection itself can affect prognosis remains to be elucidated (Morton et al. 2006).

In the present study, we performed sentinel lymph node biopsy of the left superficial inguinal lymph nodes using an isotope. We were able to omit systematic lymph node dissection by confirming the absence of inguinal lymph node metastasis (-) through rapid pathological diagnosis.

Postoperative adjuvant therapy is the treatment of choice in patients with regional lymph node metastasis and primary malignant tumors of the skin larger than 4 mm. Currently, DAV-Feron therapy (DTIC/ACNU/VCR combination therapy with interferon β injection at the site of the operative wound) is the standard therapy for melanoma internationally. A multi-center trial investigated the effectiveness of adjuvant therapy after radical operations for previous-UICC stage II and III patients (thickness >4 mm or positive regional lymph nodes) in Japan. The results showed that compared with historical controls treated with DAV only, the combined therapy significantly improved the 5-year survival rate (65.1% vs. 46.2%; P < 0.05) (Yamamoto & Ishikawa 1996).

We did not detect lymph node metastasis in the present patient; however, we selected DAV-Feron therapy because the tumor was larger than 4 mm.

Other therapies for melanomas with distal metastasis currently include high-dose IL-2 therapy and molecular targeted drugs.

High-dose IL-2 therapy has a response rate of approximately 15% and a complete response rate of 6%; however, it is associated with severe adverse reactions and is therefore not considered beneficial. Nevertheless, long-term, relapse-free survival was reported in a low percentage of patients (approximately 5%) (Atkins et al. 1998).

The molecular targeted drug BAY 43-9006 (sorafenib), a Raf-1 inhibitor, is noteworthy. In addition to inhibiting the MAPK signaling pathway, this drug also inhibits tyrosine kinase receptors associated with angiogenesis such as VEGFR-2, VEGFR-3, PDGFR- β, Flt-3, and c-KIT. In a phase I/II clinical trial, sorafenib combined with carboplatin/paclitaxel achieved impressive results with a partial response rate of 40% and a stable disease (SD) rate of 43%. A phase III clinical trial is currently underway (Strumberg 2005).

## Conclusions

The prognosis of mucous membrane-derived malignant melanomas, including those in the vaginal wall, is poor compared to those derived from the skin. Currently, surgical resection has the highest probability of improving the prognosis of patients when used as initial treatment for Stage I disease. By combining treatment with sentinel lymph node biopsy, we were able to accurately determine the stage of disease and thus avoid systematic lymph node dissection and further surgical treatments. We anticipate that post-operative adjuvant DAV-Feron therapy could be combined with molecular targeted drugs such as sorafenib in the future.

## Consent

Written informed consent was obtained from the patient for the publication of this report and any accompanying images.
